# Integrated GWAS and Transcriptome Analysis to Identify Genes Underlying Plant Height and Ear Height Plasticity in Maize Germplasm

**DOI:** 10.3390/genes16101225

**Published:** 2025-10-16

**Authors:** Liang Tu, Pengfei Liu, Angui Wang, Dong Wang, Yunfang Zhu, Gang Li, Yulin Jiang, Xun Wu, Zhiming Zhang, Zehui Chen, Xiangyang Guo

**Affiliations:** 1 Institute of Upland Food Crops, Guizhou Academy of Agricultural Sciences, Guiyang 550006, China; dytu1011@163.com (L.T.); chenzh907@sina.com (Z.C.); 2 State Key Laboratory of High-Efciency Production of Wheat-Maize Double Cropping, The Shennong Laboratory, Henan Agricultural University, Zhengzhou 450002, China; 3 Biotechnology Research Institute, Fujian Academy of Agricultural Sciences, Fuzhou 350003, China; 4 State Key Laboratory of Crop Biology, College of Life Sciences, Shandong Agricultural University, Taian 271018, China

**Keywords:** temperate–tropical maize, phenotypic plasticity, genetic mining

## Abstract

Background: Integrating tropical and temperate maize germplasm represents a pivotal strategy for driving genetic innovation, with phenotypic plasticity serving as a critical indicator of successful integration. Methods: We analyzed 155 inbred lines across 5 contrasting environments to investigate variation and plasticity in plant height (PH) and ear height (EH). Results: Genome-wide association studies (GWAS) identified 77 loci associated with phenotypic plasticity, while the transcriptome profiling of Mo17 and T32 in Zhangye and Sanya revealed 9 candidate genes potentially regulating these traits, including *Zm00001d043110*, *Zm00001d053972*, and *Zm00001d010154*. Conclusions: These results provide valuable genetic resources and theoretical foundations for germplasm improvement. The identified genes hold promise for molecular marker development, facilitating the breeding of elite lines with stable PH and EH across environments, thereby contributing to maize improvement and enhancing global food security.

## 1. Introduction

As one of the world’s most important cereal crops, maize is paramount for ensuring global food security [1]. China has a vast territory with extensive corn-growing regions [2]. Due to the influence of climatic conditions, topographical features, and farming systems, the maize varieties suitable for different ecological regions vary significantly [3]. With the increasing uncertainty of climate change, widespread climate diversity, and extreme weather events triggered by global warming, maize production is facing unprecedented challenges [4,5]. At the same time, issues such as a narrow genetic base of germplasm and a single model of hybrid vigor utilization have become increasingly prominent, restricting innovation in maize breeding and production development. To address these challenges, there is an urgent need to explore new germplasm and establish novel hybrid vigor models [6]. Tropical maize possesses rich genetic diversity, strong resistance, and significant general combining ability (GCA), providing an important resource for expanding the genetic base of maize in China and supporting the breeding of drought-resistant varieties and strong hybrid combinations [7]. However, the strong light cycle sensitivity and vigorous vegetative growth characteristics of tropical maize can lead to prolonged growing periods, excessive plant elongation, and reduced yields in temperate regions, severely limiting its direct application in these areas [8]. Therefore, elucidating the mechanisms of phenotypic plasticity in tropical maize could provide scientific guidance for breeders to precisely improve temperate–tropical maize and develop self-pollinated lines suitable for both northern and southern regions.

While maize is known for its broad ecological adaptability, the introduction of locally adapted varieties into a dissimilar ecological zone can cause pronounced alterations in phenotypic traits such as plant structure and yield, with the potential for considerable financial detriment [9]. For example, when tropical maize is introduced into temperate regions, its plant height (PH) and ear height (EH) may increase significantly, with some plants reaching heights of around 5 m. Moreover, research indicates a significant positive correlation between maize plant height and ear height [10,11]. Building on this, extensive research efforts have been dedicated to studying maize plant architecture within the framework of germplasm utilization and domestication [10,12,13,14].

Phenotypic plasticity refers to the phenomenon where an individual with the same genotype exhibits different phenotypic expressions in different environmental conditions [15,16]. It is a core manifestation of the ecological adaptability of crops [17]. In the improvement of temperate–tropical maize germplasm, reducing phenotypic plasticity is an important goal of artificial selection and a key strategy for developing self-pollinated lines suitable for both northern and southern regions.

Previous studies have demonstrated that crop adaptation is shaped by complex genetic regulatory networks, with key genes playing pivotal roles in this process [18]. Much of the existing research has focused on the genetic and molecular mechanisms underlying crop adaptation, often using flowering time as a primary indicator of phenotypic plasticity [19]. However, examining the environmental adaptation of other traits, particularly PH and EH, is equally important [20]. PH and EH represent some of the most prominent phenotypic differences in tropical maize when grown in temperate regions. Their stability not only reflects the adaptability of the materials but also serves as a key reference for evaluating the success of temperate–tropical maize germplasm improvement. Nonetheless, research on the phenotypic plasticity of PH and EH remains relatively limited.

In this study, a total of 155 inbred lines were selected as experimental materials. These materials were cultivated across five distinct environments: Sanya, Hainan; Guiyang, Guizhou; Shilin, Yunnan; Mojiang, Yunnan; and Zhangye, Gansu, to systematically investigate their phenotypic performance in terms of PH and EH, and the plasticity of these two traits was evaluated. Based on the genotypic data of these inbred lines, a genome-wide association study (GWAS) was performed to identify genetic loci associated with trait plasticity. Furthermore, by integrating transcriptome data of the inbred lines Mo17 and T32 from the Zhangye (Gansu) and Sanya (Hainan) environments, candidate genes controlling the plasticity of PH and EH were further screened. The results of this study will provide valuable genetic resources for developing self-pollinated lines suitable for both northern and southern regions and lay a solid theoretical foundation for further research on the broad adaptability mechanisms of maize.

## 2. Materials and Methods

### 2.1. Experimental Materials and Field Assessment

This study used 155 inbred lines as experimental materials, representing a diverse genetic background, including tropical germplasm (e.g., Tuxpeño, Suwan), temperate germplasm (e.g., Lancaster, Iodent), and temperate–tropical improved germplasm.

The panel materials were planted and phenotyped in 2020 at three locations: Guiyang (26.5° N, 106.7° E) in Guizhou Province, Sanya (18.36° N, 109.16° E) in Hainan Province, and Zhangye (38.93° N, 100.45° E) in Gansu Province. In 2022, the trials were conducted in Shilin (24.66° N, 103.38° E) and Mojiang (23.46° N, 101.67° E) in Yunnan Province. Each line was grown in a single 3 m row containing 12 individual plants. Fertilization, irrigation, pest control, and weed management followed local standard field protocols for all trials.

### 2.2. Genotype Identification

Genotype identification was carried out by the research team in 2019 [21]. The procedure was as follows: These leaves were collected from the experimental materials at the five-leaf stage. Genomic DNA was extracted using a modified CTAB method, and its integrity and concentration were assessed by agarose gel electrophoresis and spectrophotometric analysis. DNA samples that met the quality standards were then sent to Beijing Compson Biotechnology Co., Ltd. (Beijing, China). for subsequent genotype analysis. Genotyping was performed using the Maize SNP 50 chip, which contains 60,000 SNP markers. The SNP data were processed, and high-quality loci were selected according to the method described by Wu et al. [21].

### 2.3. Quantitative Evaluation of Phenotypic Plasticity

At 15 days after pollination, plant height and ear height of the maize population were measured. Phenotypic plasticity of these traits was assessed using the Finlay–Wilkinson regression model [22]:

*Y_ij_* = *μ* + *G_i_* + *E_j_* + *β_i_E_j_* + *ε_ij_*

where *Y_ij_* is the phenotypic value of the *i*-th genotype in the *j*-th environment, *μ* is the grand mean, *G_i_* and *E_j_* represent the effects of genotype and environment, respectively, and *β_i_* is the regression coefficient of the *i*-th genotype, reflecting its sensitivity to environmental variation and thus its plasticity. The interaction term *β_i_E_j_* captures genotype–environment interactions, while *ε_ij_* denotes residual error.

Quantifying phenotypic plasticity provides a framework to assess the adaptive capacity of maize genotypes. It facilitates the identification of lines with broad environmental adaptability as well as those with environment-specific advantages, thereby informing both ecological studies and breeding strategies.

### 2.4. Genotype-Phenotype Association Analysis

A total of 40,648 SNPs were employed to perform genome-wide association analyses of phenotypic traits and genotypic variation using the GAPIT package in R (v4.4.0) with the FarmCPU model [23]. A significance threshold of LOD > 3.0 was applied to identify trait-associated loci. This approach ensured that the detected loci represented robust associations, thereby providing a reliable basis for subsequent candidate gene identification and functional analysis.

### 2.5. Transcriptome Data Analysis

Transcriptome sequencing was conducted as previously described [24]. Two maize inbred lines, T32 and Mo17, were cultivated at two experimental sites, Sanya and Zhangye, which represent the most geographically distant locations in this study and exhibit pronounced differences in climatic conditions. Leaf samples were collected at the V9 growth stage from three biological replicates per line. Total RNA was extracted using TRIzol reagent, and cDNA library preparation and RNA sequencing were performed by Biomarker Technologies (Beijing, China) on the Illumina HiSeq 2000 platform. High-quality clean reads were aligned to the maize B73 reference genome (assembly V4) using TopHat2 [25]. Gene expression levels were quantified as FPKM (fragments per kilobase of transcript per million mapped reads). Differential expression analysis was conducted using the DESeq package in R, with significantly differentially expressed genes identified based on a threshold of Padj < 0.05 and |log2 (fold change)| ≥ 1 [26]. This experimental design enabled the evaluation of transcriptomic responses under contrasting environmental conditions, thereby providing insights into genotype–environment interactions at the molecular level.

### 2.6. Candidate Gene Prediction

Using the maize B73 reference genome (version V4), genes located within ±100 kb of significant SNPs were designated as candidate genes associated with phenotypic plasticity. Functional annotations of these genes were subsequently retrieved from multiple bioinformatics databases. This approach enables the systematic identification of potential functional genes and provides a foundation for dissecting the molecular mechanisms underlying phenotypic variation.

## 3. Results

### 3.1. Plasticity Analysis of PH and EH

**Figure 1:**
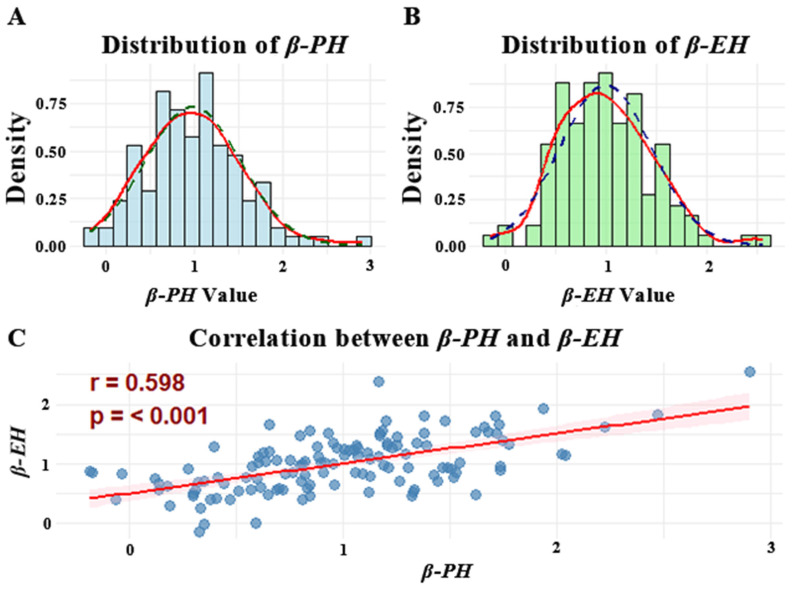
Normal distribution and correlation analysis of plasticity in PH and EH across the population. (**A**) Phenotypic plasticity for plant height (*β-PH*) exhibited an approximately normal distribution; (**B**) Phenotypic plasticity for ear height (*β-EH*) followed a near-normal distribution; (**C**) Correlation analysis between plant height and ear height phenotypic plasticity revealed a statistically significant relationship (*p* < 0.001). The dashed lines in (**A**,**B**) represent the theoretical normal distribution curves, while the solid lines depict the normal distribution curves fitted to the empirical data. The red solid line in (**C**) indicates the correlation between *β-PH* and *β-EH*.

Using the Finlay–Wilkinson regression model, we analyzed the plasticity of PH and EH in the panel. The results revealed distinct environmental sensitivity among lines such as LH123, 81162, and Mo17 exhibited relatively low sensitivity of PH and EH to environmental variation, whereas T32, QB2827, and QB2828 displayed pronounced environmental sensitivity. For instance, T32 showed the highest *β-PH* and *β-EH* values, 2.90 and 2.55, respectively. As illustrated in Figure 1, the probability density curves of PH and EH plasticity followed a bell-shaped pattern, with low frequencies at both extremes and higher frequencies in the middle, consistent with a normal distribution. The ranges of plasticity values for PH and EH were −0.18~2.90 and −0.14~2.55, respectively. Moreover, the two traits were highly significantly correlated, with a correlation coefficient of 0.598 (Figure 1C).

### 3.2. GWAS Analysis

**Figure 2:**
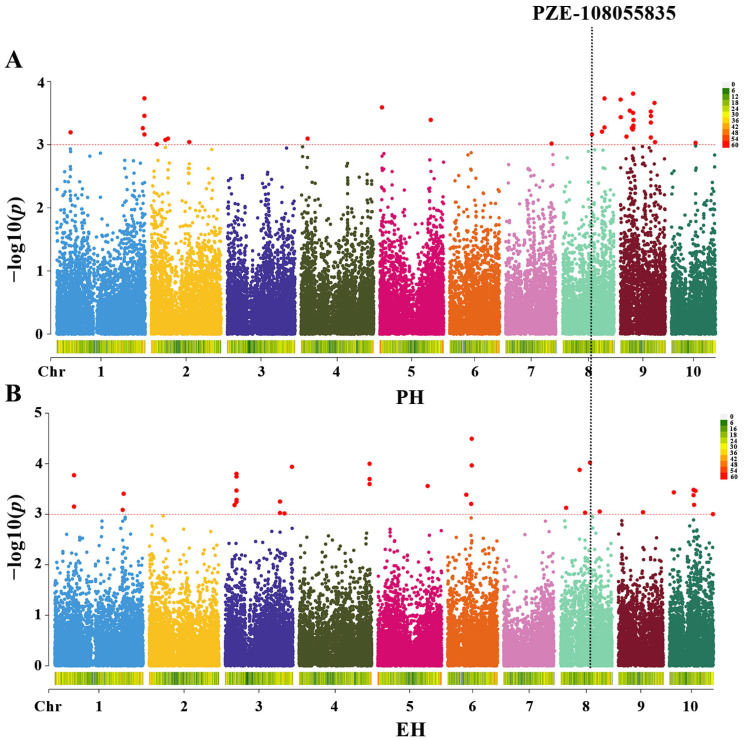
GWAS results for plasticity of PH and EH. (**A**) indicates loci with LOD > 3.0 for phenotypic plasticity of plant height (*β-PH*) in the GWAS results; (**B**) indicates loci with LOD > 3.0 for phenotypic plasticity of ear height (*β-EH*) in the GWAS results; the vertical dashed line signifies the co-localization of both traits at the PZE-108055835 locus.

We conducted a GWAS for PH and EH plasticity using the FarmCPU model and identified 77 loci significantly associated with trait plasticity (Figure 2; Supplementary Table S1). Of these, 41 loci were linked to PH plasticity (Figure 2A), distributed across chromosome 1 (5 loci), chromosome 2 (5), chromosome 4 (1), chromosome 5 (2), chromosome 7 (1), chromosome 8 (4), chromosome 9 (22), and chromosome 10 (1). In contrast, 35 loci were associated with EH plasticity (Figure 2B), located on chromosome 1 (4 loci), chromosome 3 (11), chromosome 4 (3), chromosome 5 (1), chromosome 6 (4), chromosome 8 (5), chromosome 9 (1), and chromosome 10 (6).

**Figure 3:**
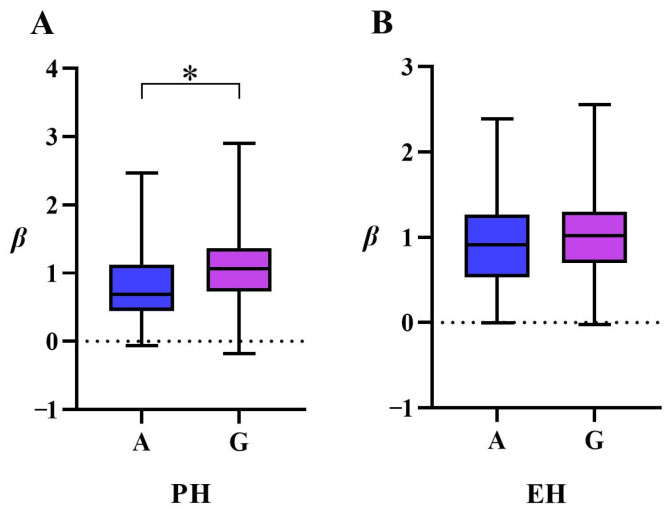
Superior allelic variant at the PZE-108055835. (**A**) illustrates the allelic variation in *β-PH* at locus PZE-108055835, showcasing a significant difference; (**B**) shows the allelic variation in *β-EH* at the same locus, which was not statistically significant (*, Student’s *t*-test, *p* < 0.05).

A comparative analysis of the GWAS results revealed a co-localized locus, PZE-108055835, on chromosome 8. Allelic variation analysis indicated a G/A polymorphism at this locus: 107 accessions carried the G allele and 41 carried the A allele. Phenotypic effect analysis further demonstrated that lines with the G allele exhibited reduced plasticity in both PH (*β-PH*) and EH (*β-EH*) compared with those carrying the A allele, with mean reductions of 0.22 and 0.11, respectively. The difference in *β-PH* was statistically significant (*p* < 0.05) (Figure 3). Collectively, these findings indicate that the A allele represents a superior variant at the PZE-108055835 locus and may serve as a valuable genetic resource for improving phenotypic plasticity and enhancing adaptability in maize breeding programs.

### 3.3. Integrating GWAS and Transcriptomics to Predict Candidate Genes

**Figure 4:**
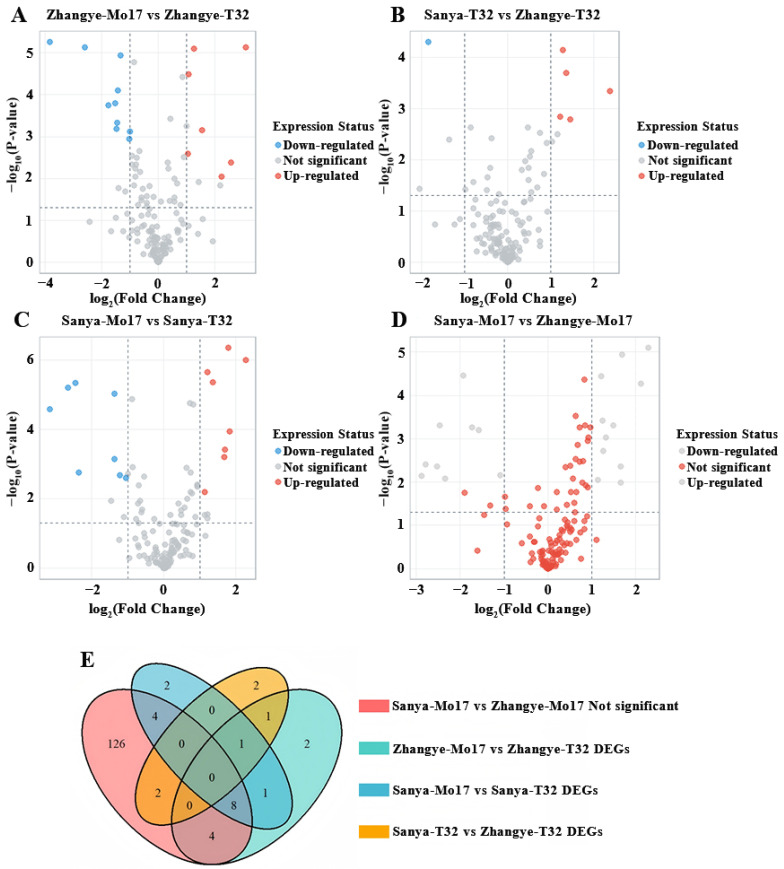
Expression patterns of 164 candidate genes in Mo17 and T32 across Zhangye and Sanya. (**A**) Volcano plot of Zhangye-Mo17 vs. Zhangye-T32, Blue represents upregulated genes and red represents downregulated genes. (**B**) Volcano plot of Sanya-T32 vs. Zhangye-T32, Blue represents upregulated genes and red represents downregulated genes. (**C**) Volcano plot of Sanya-Mo17 vs. Sanya-T32, Blue represents upregulated genes and red represents downregulated genes. (**D**) Volcano plot of Sanya-Mo17 vs. Zhangye-Mo17, Red indicates genes with no significant difference. (**E**) Venn diagram of “Sanya-Mo17 vs. Zhangye-Mo17 Not significant”, “Zhangye-Mo17 vs. Zhangye-T32 DEGs”, “Sanya-Mo17 vs. Sanya-T32 DEGs,” and “Sanya-T32 vs. Zhangye-T32 DEGs.”.

By integrating data from the MaizeGDB (https://maizegdb.org, accessed on 13 September 2025) and the maize B73 reference genome (RefGen V4), we identified 164 candidate genes associated with the plasticity of PH and EH (Supplementary Table S2). Transcriptome analysis revealed 17 differentially expressed genes (DEGs) between Mo17 (with lower *β-PH* and *β-EH*) and T32 (with higher *β-PH* and *β-EH*) in Zhangye, including 10 downregulated and 7 upregulated genes (Figure 4A). In Sanya, 16 DEGs were identified between the two lines, comprising 8 downregulated and 8 upregulated genes (Figure 4C). Comparative expression profiling of the 164 candidate genes across environments showed that T32 exhibited 6 DEGs between Sanya and Zhangye, including 1 downregulated and 5 upregulated genes (Figure 4B). In contrast, Mo17, which maintained relatively stable PH and EH across environments, showed no significant expression changes for 144 genes between the two sites (Figure 4D).

A comparative analysis of candidate genes identified from four contrast groups—“Sanya-Mo17 vs. Zhangye-Mo17 Not significant”, “Zhangye-Mo17 vs. Zhangye-T32 DEGs”, “Sanya-Mo17 vs. Sanya-T32 DEGs”, and “Sanya-T32 vs. Zhangye-T32 DEGs”—was conducted. A Venn diagram (Figure 4E) revealed 9 candidate genes shared among three comparison groups (Table 1). Functional annotation indicated that these genes are involved in critical processes related to plant growth, development, and environmental responses. For instance, *Zm00001d043110* was consistently downregulated in Mo17 relative to T32 in both Zhangye and Sanya, whereas T32 displayed upregulation of this gene in Sanya compared with Zhangye. This gene encodes Beta-glucosidase 11 (https://www.uniprot.org, accessed on 13 September 2025), which likely participates in cell wall formation and degradation, hormone activation, and responses to both biotic and abiotic stresses.

**Figure 5:**
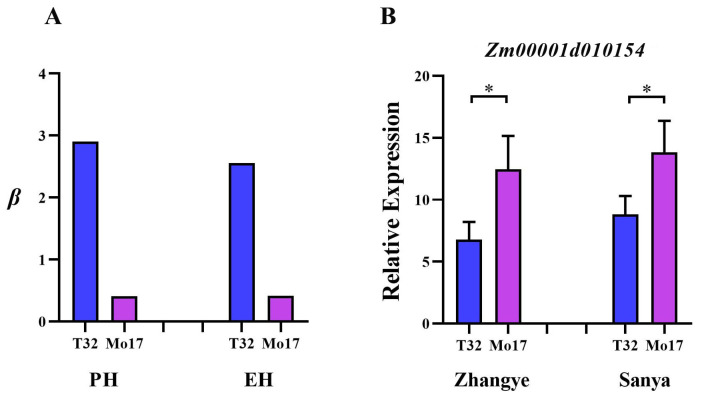
Relative expression of *Zm00001d010154* across environments. (**A**) Differences in *β-PH* (left) and *β-EH* (right) between T32 and Mo17; (**B**) Relative expression level of *Zm00001d010154* in T32 and Mo17 under Zhangye (left) and Sanya (right) environments; *, Student’s *t*-test, *p* < 0.05.

An in-depth analysis of the co-localized locus PZE-108055835 identified *Zm00001d010154* as a key candidate gene. Transcriptome profiling showed that this gene was differentially expressed between T32 (with higher *β-PH* and *β-EH*) and Mo17 (with lower *β-PH* and *β-EH*) across both the Sanya and Zhangye environments (Figure 5), highlighting its potential role in regulating phenotypic plasticity.

## 4. Discussion

Phenotypic plasticity is a critical indicator for evaluating the success of temperate–tropical germplasm integration. In this study, we employed the Finlay–Wilkinson regression model to assess the plasticity of PH and EH in 155 maize inbred lines. The results showed that T32 exhibited high plasticity in both PH and EH, reflecting its strong sensitivity to environmental variation. Previous research has also demonstrated that T32, as a photoperiod-sensitive tropical line, shows substantial environmental responses in traits such as plant architecture and flowering time [24,27,28], further confirming the effectiveness and reliability of the Finlay–Wilkinson model in plasticity analysis. In contrast, Mo17 and other temperate lines displayed relatively stable PH and EH across environments, suggesting reduced sensitivity to environmental fluctuations. This finding highlights the potential of temperate germplasm to address the challenge of excessive PH and EH often observed when tropical germplasm is introduced into temperate regions. Building on this observation, we developed a Suwan–Lancaster population by crossing Mo17 (Lancaster) with Suwan germplasm and subsequently identified a subset of temperate–tropical inbred lines with stable performance for PH and EH across multiple environments [29]. These results not only provide a solid germplasm foundation for improving PH- and EH-related traits but also offer theoretical support for the integration and genetic improvement of temperate–tropical maize germplasm.

The identification of broad-adaptation/phenotypic plasticity genes is of great importance for maize breeding [30,31]. By integrating GWAS and transcriptome data, we predicted nine candidate genes potentially associated with PH and EH plasticity. Functional annotation indicated that these genes play important roles in regulating flowering time, plant height, and stress resistance, highlighting their roles in modulating plant responses to environmental variation. For example, *Zm00001d003607* and *Zm00001d044542*, both encoding F-box domain-containing proteins (https://www.uniprot.org, accessed on 13 September 2025), have been previously reported to regulate flowering time and resistance to banded leaf and sheath blight in maize [32,33], underscoring their importance in environmental response. Notably, the expression of these genes showed no significant differences in Mo17 (with lower *β-PH* and *β-EH*) across Zhangye and Sanya; however, both were downregulated in T32 (with higher *β-PH* and *β-EH*) relative to Mo17, suggesting their potential roles in regulating PH and EH plasticity.

Similarly, *Zm00001d053972* showed stable expression in Mo17 across environments but was significantly upregulated in T32. This gene encodes a DNAJ heat shock family protein (https://www.uniprot.org, accessed on 13 September 2025), a stress-responsive factor widely present in plant cells, which is involved in diverse biotic and abiotic stress responses. Luo et al. [34] reported that such proteins can modulate gibberellin (GA) homeostasis, thereby influencing plant architecture in rice. In addition, we identified a locus, PZE-108055835, that simultaneously regulates PH and EH plasticity, and further pinpointed *Zm00001d010154* as its associated candidate gene. Transcriptome analysis revealed significant differential expression of this gene between T32 (with higher *β-PH* and *β-EH*) and Mo17 (with lower *β-PH* and *β-EH*) across Zhangye and Sanya, indicating that it is also a strong candidate gene underlying plasticity.

The discovery of these candidate genes provides not only theoretical insights into the plasticity of PH and EH but also practical guidance for integrating and improving temperate–tropical maize germplasm. These genes represent promising targets for developing molecular markers, which can facilitate marker-assisted selection (MAS) to identify and breed superior temperate–tropical inbred lines with stable PH and EH across diverse environments. Such advances will accelerate germplasm innovation and contribute to ensuring national food security.

## 5. Conclusions

In summary, this study establishes phenotypic plasticity as a robust framework for evaluating tropical–temperate maize germplasm integration. By identifying variation across environments and integrating GWAS with transcriptome analyses, we identified 77 loci and 9 candidate genes that deepen our understanding of the genetic basis underlying plant height and ear height plasticity. These findings provide a theoretical foundation for understanding the genetic architecture of phenotypic plasticity and enrich the conceptual basis for germplasm innovation, thereby advancing our knowledge of genotype-by-environmental interactions in shaping complex agronomic traits in maize.

## Figures and Tables

**Table 1:** Candidate genes identified by integrating GWAS and transcriptome analysis.

Gene	Comparison	Difference Expressed	Function
*Zm00001d003607*	Sanya-Mo17 vs. Zhangye-Mo17	No	F-box domain-containing protein
	Zhangye-Mo17 vs. Zhangye-T32	Down	
	Sanya-Mo17 vs. Sanya-T32	Down	
*Zm00001d023682*	Sanya-Mo17 vs. Zhangye-Mo17	No	ABC transporter F family member 1
	Zhangye-Mo17 vs. Zhangye-T32	Up	
	Sanya-Mo17 vs. Sanya-T32	Up	
*Zm00001d026647*	Sanya-Mo17 vs. Zhangye-Mo17	No	DNA mismatch repair protein MSH3
	Zhangye-Mo17 vs. Zhangye-T32	Down	
	Sanya-Mo17 vs. Sanya-T32	Down	
*Zm00001d026651*	Sanya-Mo17 vs. Zhangye-Mo17	No	Expressed protein%3B protein
	Zhangye-Mo17 vs. Zhangye-T32	Up	
	Sanya-Mo17 vs. Sanya-T32	Up	
*Zm00001d043110*	Zhangye-Mo17 vs. Zhangye-T32	Down	Beta-glucosidase 11
	Sanya-Mo17 vs. Sanya-T32	Down	
	Sanya-T32 vs. Zhangye-T32	Up	
*Zm00001d044542*	Sanya-Mo17 vs. Zhangye-Mo17	No	F-box domain-containing protein
	Zhangye-Mo17 vs. Zhangye-T32	Down	
	Sanya-Mo17 vs. Sanya-T32	Down	
*Zm00001d045384*	Sanya-Mo17 vs. Zhangye-Mo17	No	Superoxide dismutase16
	Zhangye-Mo17 vs. Zhangye-T32	Down	
	Sanya-Mo17 vs. Sanya-T32	Down	
*Zm00001d045386*	Sanya-Mo17 vs. Zhangye-Mo17	No	Ethylene-responsive transcription factor RAP2-2
	Zhangye-Mo17 vs. Zhangye-T32	Down	
	Sanya-Mo17 vs. Sanya-T32	Down	
*Zm00001d053972*	Sanya-Mo17 vs. Zhangye-Mo17	No	DNAJ heat shock family protein
	Zhangye-Mo17 vs. Zhangye-T32	Up	
	Sanya-Mo17 vs. Sanya-T32	Up	

## Data Availability

The original contributions presented in this study are included in the article/Supplementary Materials. Further inquiries can be directed to the corresponding author(s).

## Supplementary Materials

The following supporting information can be downloaded at https://www.mdpi.com/article/10.3390/genes16101225/s1, Table S1: The 77 loci significantly associated with trait plasticity; Table S2: The 164 candidate genes associated with the plasticity of PH and EH.

## Abbreviations

The following abbreviations are used in this manuscript:

PH	Plant height
EH	Ear height
GWAS	Genome-wide association studies
*β-PH*	Plasticity values of plant height
*β-EH*	Plasticity values of ear height
FPKM	Fragments per kilobase of transcript per million mapped reads
DEGs	Differentially expressed genes
GCA	Significant general combining ability
